# Draft genome sequences of three *Bacillus* spp. from the University of the Philippines Culture Collection

**DOI:** 10.1128/mra.01471-25

**Published:** 2026-08-17

**Authors:** Kyla Carmea L. Javier, Paul Christian T. Gloria, Maria Auxilia T. Siringan, Vina B. Argayosa

**Affiliations:** 1 Microbiological Research and Services Laboratory, Natural Sciences Research Institute, University of the Philippines Diliman 54727, Quezon City, Philippines; 2 Department of Science and Technology, Philippine Science High School Main Campus in Diliman, Quezon City, Philippines; University of Wisconsin-Madison, Madison, Wisconsin, USA

**Keywords:** *Bacillus*, *Bacillus cereus*, *Bacillus velezensis*, stress tolerance, plant-growth promotion, secondary metabolite production, UPCC

## Abstract

This study reports the assembled and annotated draft genomes of three *Bacillus* spp. isolates from the University of the Philippines Culture Collection. Genome analysis predicted genes for stress tolerance, plant-growth promotion, and secondary metabolite production, highlighting their potential for diverse biotechnological applications in agriculture and natural product synthesis.

## ANNOUNCEMENT

*Bacillus* spp. have shown capabilities in various applications such as plant growth promotion and secondary metabolite production (1–6). Two *Bacillus* spp. isolates from the University of the Philippines Culture Collection (UPCC) (WDCM 310), maintained at 6°C as lyophilized cultures since 1980, remain unexplored. *Bacillus megaterium* UPCC 1088 (6th edition)/UPCC 88 (5th edition) purchased from the Micro-Biological Laboratory, Pasay City (14.553565349949126, 121.00018726572775); *Bacillus* sp. UPCC 1222 (6th edition)/UPCC 222 (5th edition) isolated through settle plates at the University of Santo Tomas, Manila (14.609992238937048, 120.98958338520913) (7, 8); and *Bacillus* sp. UPCC 1445 (pre-deposition identifier: 23W112) from a pellicle in a Lauryl Sulfate Broth (Merck, Darmstadt, Germany) culture from a water sample from Makati City (14.532480097505955, 121.0218937951251) are reported in this work. The three organisms are maintained in Nutrient Agar (Merck, Darmstadt, Germany) plates at 35°C. We sequenced these isolates to confirm their identities and predict their potential applications based on stress tolerance, plant growth-promoting, and biosynthetic genes.

**Fig 1:**
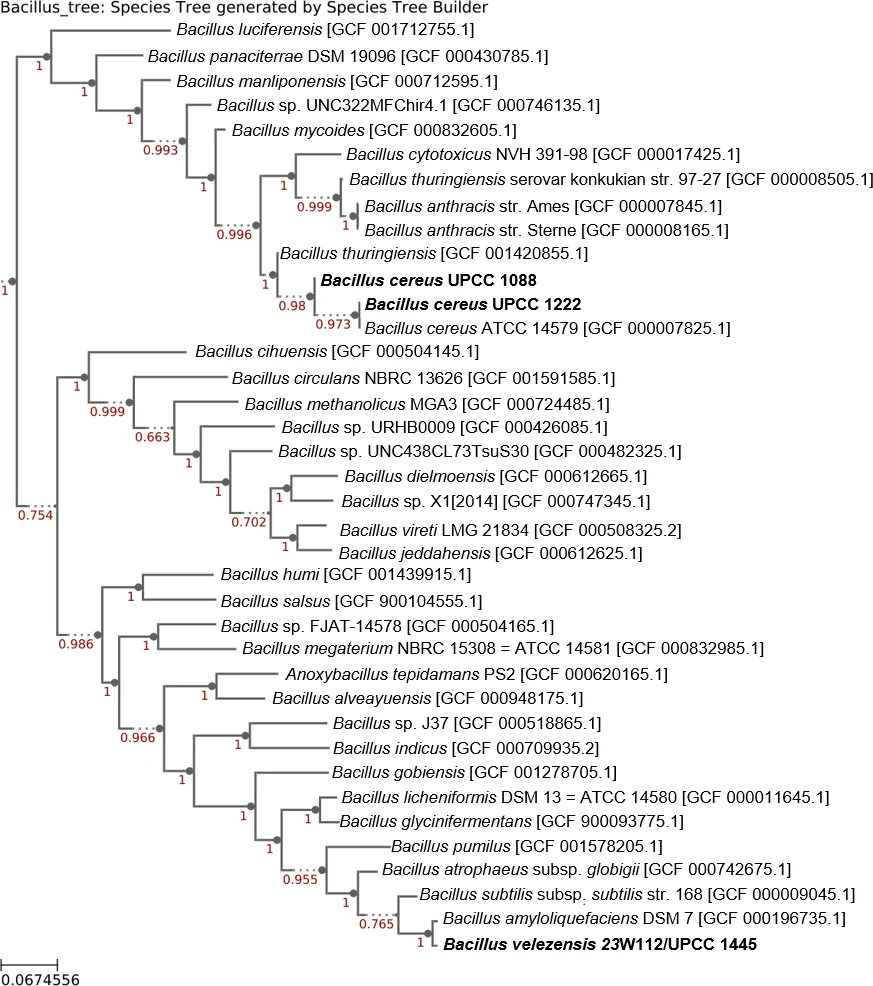
Species tree of UPCC *Bacillus* spp. isolates generated through SpeciesTree v.2.2.0 using a set of 49 core, universal genes defined by COG gene families. The List of Prokaryotic Names with Standing in Nomenclature lists *B. velezensis* as a heterotypic synonym of *Bacillus amyloliquefaciens* subsp. *plantarum* thus the clustering of the two organisms in the species tree (18, 19).

The isolate DNA was extracted from pure Nutrient Broth (Merck, Darmstadt, Germany) cultures using the NucleoSpin Microbial DNA kit (Macherey-Nagel, Düren, Germany) according to manufacturer’s protocol and sent to Macrogen (South Korea) for sequencing. Library preparation was done using the TruSeq Nano DNA kit (Illumina Inc., California, USA), and sequencing was performed using an Illumina NovaSeq 6000 (Illumina Inc., California, USA) platform under a 150-bp paired-end setting to a throughput of 2 Gb per sample. The following bioinformatics tools were used in default settings unless otherwise stated. Raw reads were quality-trimmed to Q30 using Trimmomatic v.0.36 (9), and trimmed reads were assembled using Unicycler v.0.5 (10) and evaluated using QUAST v.5.2.0 (11). Genome completeness and contamination were checked using BUSCO v.5.5 (12) and CheckM v.1.2. (13). Taxonomic identities were established using GTDB-Tk v.1.7.0 and JSpeciesWS (14). A species tree (Fig. 1) was constructed based on a set of 49 core, universal genes defined by Clusters of Orthologous Groups (COG) gene families using SpeciesTree v.2.2.0 (15). General gene annotation was performed using the National Center for Biotechnology Information (NCBI) Prokaryotic Genome Annotation Pipeline v.6.10 (16). antiSMASH 7.1.0 (17) was used to predict potential secondary metabolite biosynthetic gene clusters (smBGCs) with a relaxed detection strictness and all extra features turned on.

Table 1 shows relevant sequence data of the three *Bacillus* spp.: genome sizes (4–5.6 Mbp), contigs (45–75), *N*50 values (297.9–925.1 kb), genome completeness (99.5%–99.8%), genome contamination (0%–0.11%), and GC percentages (35% and 46.22%). The identification of UPCC 1088 and UPCC 1222 was revised as *Bacillus cereus* (average nucleotide identity [ANI] = 98.66% and 99.14%, respectively), and UPCC 1445 as *Bacillus velezensis* (ANI = 98.75%). The number of predicted genes was 3,997–5,657 genes. Plant growth-promoting genes involved in nitrogen (16–20 genes) and phosphate metabolism (10–23 genes), siderophore production (23–54 genes), and stress response (41–44 genes) were predicted in all three genomes. The most abundant predicted smBGCs among the three genomes are non-ribosomal peptide synthetase, post-translationally modified peptide product-like, and trans-AT-polyketide synthase. These predicted genes involved highlight the potential use of the UPCC *Bacillus* isolates in various applications (20–22).

**TABLE 1:** Genome assembly details and BGC prediction of *Bacillus* spp. from the UPCC

Strain	Source	GTDB-tk species identification	Assembly accession no.	BioSample accession no.	SRA accession no.	GenBank accession no.	Coverage (X)	No. of contigs	Total no. of reads before assembly (bp)	Total bases (bp)	Draft genome size (Mb)	BUSCO completeness	CheckM completeness	CheckM contamination	GC content (%)	No. of CDS	No. of RNAs	L50	N50 (kb)	Number of biosynthetic gene clusters
UPCC 1088	Micro-biological Laboratory	*B. cereus*	GCF_047607505.1	SAMN45887305	SRX27118688	JBLKPE000000000.1	981	75	17,209,172	2,598,584,972	5.3	99.5	99.43	0.11	35	5,323	84	6	320.5	16
UPCC 1222	Air	*B. cereus*	GCF_047607525.1	SAMN45887306	SRX27118689	JBLKPD000000000.1	908	45	16,844,556	2,543,527,956	5.6	99.8	99.43	0.00	35	5,657	81	3	925.1	12
UPCC 1445	Tap water	*B. velezensis*	GCF_047607545.1	SAMN45887307	SRX27118690	JBLKPC000000000.1	1412	70	18,708,578	2,824,995,278	4	99.8	99.59	0.00	46.22	3,997	79	5	297.9	20

## Data Availability

Raw reads, genome assemblies, and Prokaryotic Genome Annotation Pipeline genome annotation can be accessed in NCBI (Bioproject ID PRJNA1199749). Other genome annotation files and supplementary materials can also be found in Zenodo (https://doi.org/10.5281/zenodo.16810166).
